# Bravery

**Published:** 1862-11

**Authors:** 


### BRAVERY.

Every man thinks himself brave until the battle. We have long been under the impression that we feared nothing and nobody, in spite of some dreamy intimations now and then. It is generally supposed, that, as a man acts in his dreams, he would act under the same circumstances if awake. By this rule, we are neither one thing nor the other, but rather, if anything, the other, as to bravery. We have frequently dreamed of battles, and invariably there was a contest within, and a question—a dilemma with three horns, and all sharp. We wanted to run away, but was afraid of the disgrace; we wanted to fight, for the credit of being considered brave, but were afraid of the bullet; and we wanted to be *statu quo*, and thus be on the safe side both ways! Once we were fairly driven "to the scratch," most sorely against our will, and, at the very first crack, we "hollered," and waked up, but wasn't hurt! But, not to put too fine a point upon it, nor too long, we find that, as circumstances change, we have changed with them. When the circulation of another journal barely reached hundreds a month, we wrote exactly as we thought; didn't care a straw for anybody, and took great delight in pleasing ourselves, being the principal patron of our pet. But, now that we print thousands monthly, we find ourselves backing down from our high ground, and instinctively shrink from writing a line, or even word, that would unpleasantly affect this, that, or the other "exchange;" this, that, or the other minister; this, that, or the other sect; this, that, or the other party; this, that, or the other trade, calling, or profession. We had so much rather give any one a pleasant feeling than an unpleasant one, that we are very often amazed, in looking over our exchanges—masculine, feminine, and neuter, regular, irregular, and defective—to see them pitching into each other, tooth and toe-nail, with savage sarcasm, with merciless malignity, with brutal recklessness, or cowardly innuendo. When we look at these saliences as coming from men of all creeds, confessions, and callings, and contemplate our own smoothness and milk-and-water-ness, we feel for a moment as if we were a mess of dish-water, and forthwith write a tremendous (in our own estimation) article against this doctrine, and that "pathy," and the other heresy of law, Gospel, or physic. And when the last word is written, with the fever of excitement at the boiling point, we feel ready to sit for a portrait of double-refined complacency. But when we have taken a romp with the children; or, with a little one on either knee before the flaming grate, sing with them,

"Jerusalem, my happy home,"

"There is a happy land;"

or, when we have taken dinner, or a walk into the sunshine, or played a tune on the jews-harp, and then take up the article to review at an imaginary distance of years ahead, or of a dying hour, we tear it up, and exclaim, "What's the use?" when love, and not battle, is the law of the moral universe, the weapon of Omnipotence: so that we shall endeavor to avoid writing anything for these pages in the darkness of night, in the depression of fatigue, in the dullness of a hearty meal. If we don't feel in the humor of writing, we shall wait until we do, or engage in some bustling occupation, and raise a commotion or a storm of some kind, to clear and purify the mental atmosphere. Authors, writers, editors, ministers, practice kindness to all, for it oftener converts than a kick; it oftener mends than breaks; oftener raises up than casts down; oftener elevates than degrades; oftener helps than hurts; oftener cheers than discourages; oftener wins than wounds; and, many a time, would have encouraged to saving exertions a struggling, disheartened, and despairing brother, when a harsh word, a cold look, a heartless tone, has felled him to the earth, and he has perished in his helplessness. All of woman born, be kind!

